# Virtual Screening and In Vitro Evaluation of PD-1 Dimer Stabilizers for Uncoupling PD-1/PD-L1 Interaction from Natural Products

**DOI:** 10.3390/molecules25225293

**Published:** 2020-11-13

**Authors:** Jrhau Lung, Ming-Szu Hung, Yu-Ching Lin, Chien-Hui Hung, Chih-Cheng Chen, Kuan-Der Lee, Ying Huang Tsai

**Affiliations:** 1 Department of Medical Research and Development, Chang Gung Memorial Hospital, Chiayi Branch 613, Taiwan; 2 Department of Pulmonary and Critical Care Medicine, Chang Gung Memorial Hospital, Chiayi Branch 613, Taiwan; m12049@cgmh.org.tw (M.-S.H.); lin0927@cgmh.org.tw (Y.-C.L.); 3 Department of Medicine, College of Medicine, Chang Gung University, Taoyuan 333, Taiwan; 4 Department of Respiratory Care, Chiayi Campus, Chang Gung University of Science and Technology, Chiayi 613, Taiwan; 5 Graduate Institute of Clinical Medical Sciences, Chang Gung University, Taoyuan 333, Taiwan; hungc01@mail.cgu.edu.tw; 6 Department of Hematology and Oncology, Chang Gung Memorial Hospital, Chiayi Branch 613, Taiwan; ccchen1968@gmail.com; 7 Department of Hematology and Oncology, Taipei Medical University Hospital, Taipei 110, Taiwan; kdlee@tmu.edu.tw; 8 Department of Respiratory Care, College of Medicine, Chang Gung University, Taoyuan 333, Taiwan; chestmed@cgmh.org.tw; 9 Department of Pulmonary and Critical Care Medicine, Chang Gung Memorial Hospital, Linkou Branch 333, Taiwan

**Keywords:** immunotherapy, PD-1, PD-L1, small molecular inhibitor, virtual screening

## Abstract

Genetic mutations accumulated overtime could generate many growth and survival advantages for cancer cells, but these mutations also mark cancer cells as targets to be eliminated by the immune system. To evade immune surveillance, cancer cells adopted different intrinsic molecules to suppress immune response. PD-L1 is frequently overexpressed in many cancer cells, and its engagement with PD-1 on T cells diminishes the extent of cytotoxicity from the immune system. To resume immunity for fighting cancer, several therapeutic antibodies disrupting the PD-1/PD-L1 interaction have been introduced in clinical practice. However, their immunogenicity, low tissue penetrance, and high production costs rendered these antibodies beneficial to only a limited number of patients. PD-L1 dimer formation shields the interaction interface for PD-1 binding; hence, screening for small molecule compounds stabilizing the PD-L1 dimer may make immune therapy more effective and widely affordable. In the current study, 111 candidates were selected from over 180,000 natural compound structures through virtual screening, contact fingerprint analysis, and pharmacological property prediction. Twenty-two representative candidates were further evaluated in vitro. Two compounds were found capable of inhibiting the PD-1/PD-L1 interaction and promoting PD-L1 dimer formation. Further structure optimization and clinical development of these lead inhibitors will eventually lead to more effective and affordable immunotherapeutic drugs for cancer patients.

## 1. Introduction

Immune response is tightly balanced between activation and suppression. Deviation from the balance in either direction can cause diseases, such as autoimmune disease and cancer. Genetic mutations accumulated during cancer development could generate many growth and survival advantages for cancer cells, but these mutations also mark cancer cells as targets to be eliminated by the immune system. To escape immune surveillance, cancer cells activate many immune suppressive systems to block the normal function of immune cells. It is believed that if the malfunctioned immune system can be awakened in cancer patients, deadly diseases could be cured more safely and naturally. Many attempts have been made to overcome immune suppression by modulating various co-stimulatory or co-inhibitory molecules. Currently, several successful examples, such as anti-PD-1 and anti-PD-L1 antibodies, have passed clinical trials and served patients with various cancers. It is hoped that continuous exploration and development of new strategies to overcome immune suppression could eventually improve the treatment outcome and survival for all cancer patients.

Using therapeutic antibodies is the most extensively explored strategy for overcoming tumor immune suppression due to their high specificity and strong affinity. Currently, several PD-1 blocking antibodies, such as nivolumab and pembrolizumab, have been applied to clinical practice [1,2]. These antibodies disrupt the PD-1/PD-L1 interaction by shielding the contact interface of PD-1/PD-L1. With substantial molecular interactions between these antibodies and PD-1/PD-L1, the binding affinity outcompetes the natural ligand-receptor interaction, reaching the nanomolar range and even lower [2]. Despite of these successes, these antibody drugs pose several limitations, including high production cost, low stability, immunogenicity, and poorer tissue distribution [3,4]. These have driven the search for inhibitors of a smaller size for immunotherapy. An example of these inhibitors is the high-affinity recombinant variant of the mutant PD-1 extracellular domain, which has a 45-fold increase in the binding affinity compared to its wildtype counterpart [5,6]. There is also a peptide mimetic with nanomolar potencies resembling the sequence involved in the PD-1/PD-L1 interaction according to the time-resolved fluorescence resonance energy transfer (TR-FRET) assay, the mouse splenocyte proliferation rescue assay in the presence of recombinant PD-L1 or co-cultured with PD-L1-expressing cancer cells, or IFN-γ production in a cytomegalovirus (CMV) or human immunodeficiency virus (HIV) protein-stimulated cytotoxic T lymphocyte [2,7]. Although it is generally believed that small molecules are insufficient to disrupt the protein-protein interaction, there are examples with such an ability, such as those of vincristine depolymerizing microtubules and nutalin separating MDM2 and p53 to avoid interaction [2,7]. Moreover, a few small molecular inhibitors blocking the PD-1/PD-L1 interaction have also been identified [8,9]. Nevertheless, instead of directly blocking the PD-1/PD-L1 interaction, these compounds shield the PD-1/PD-L1 interaction interface and reduce the interaction by promoting PD-L1 dimer formation [10,11,12]. This novel mechanism for blocking the PD-1/PD-L1 interaction looks promising, but further structure optimization or search for more potent ones are still required to make clinically useful small molecular PD-1/PD-L1 inhibitors become reality.

In order to seek other novel chemical structures capable of inhibiting the PD-1/PD-L1 interaction through stabilizing the PD-L1 dimer, over 180,000 chemical structures in the natural product dataset (ZBC) of the ZINC12 database were subjected to virtual screening in the current study. Two novel compounds with such an inhibitory ability were successfully identified from 111 selected candidates having a contact fingerprint similar to the known small molecular PD-1/PD-L1 inhibitor. Further structure optimization and expansion of the screening scope will eventually identify useful small molecular PD-1/PD-L1 inhibitors to help cancer patients.

## 2. Results

### 2.1. Virtual Screening of the PD-1/PD-L1 Inhibitor from Natural Products

**Figure 1:**
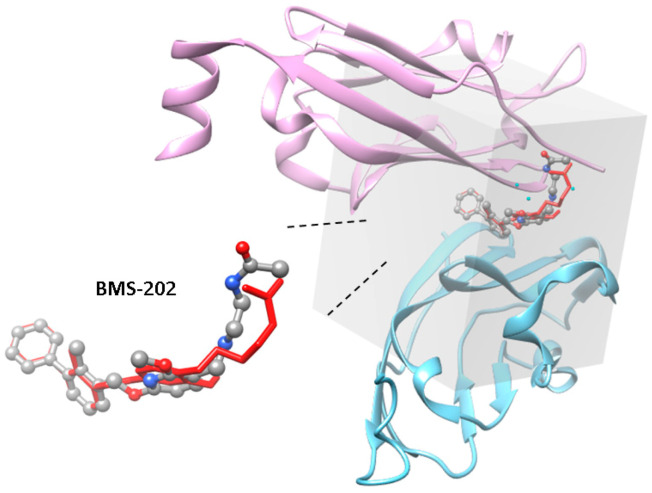
Grid box dimensions used in the virtual screening. The PD-L1 IgV domain dimer BMS-202 complex structure was fetched from the Research Collaboratory for Structural Bioinformatics Protein Data Bank (RCSB PDB) (PDB ID: 5J89). PD-L1 is illustrated with the ribbon style and labeled in plum (chain C) and light blue (chain D), respectively. Pose of BMS-202 in the crystal structure is depicted with the ball-and-stick style, in which carbon, oxygen, and nitrogen atoms are labeled in gray, red, and blue, respectively. Cocrystal water molecules in 5J89 were kept to increase the accuracy of docking and are labeled as light blue dots. The best docking pose of BMS-202 predicted by idock is depicted with the stick style and labeled in red. The image was made using UCSF Chimera [14].

To identify inhibitors capable of blocking the PD-1/PD-L1 interaction by promoting PD-L1 dimer formation from natural products, the dimer structure of the PD-L1 IgV domain (PDB ID: 5J89), and a natural product dataset, ZBC, containing 180,131 chemical structures from the ZINC12 database [13] were processed to perform virtual screening using idock. Four known PD-1/PD-L1 inhibitors, BMS-8, BMS-37, BMS-200, and BMS-202 (chemical structures shown in Figure 1), which stabilize the PD-L1 dimer [12], were also included in the screening to obtain threshold values for selecting candidates with better inhibitory potencies, and the IC50 values for BMS-8, BMS-200, and BMS-202 were reported to be 146 nM, 80 nM, and 18 nM, respectively, in their original patent document (WO2015034820). At the end of the screening, the idock values for BMS-8, BMS-37, BMS-200, and BMS-202 were −7.7, −9.16, −8.49, and −9.87 kcal/mol, respectively, and the numbers of chemical structures had idock scores less than −12, −11, −10, and the value of the most potent positive control, BMS-202, was 31, 437, 3608, and 4479, respectively. An arbitrary cutoff value slightly less than the idock score of BMS-202 was set at −9.95 kcal/mol, and 3929 chemical structures were included for downstream analysis.

### 2.2. Contact Fingerprint Analysis

**Figure 2:**
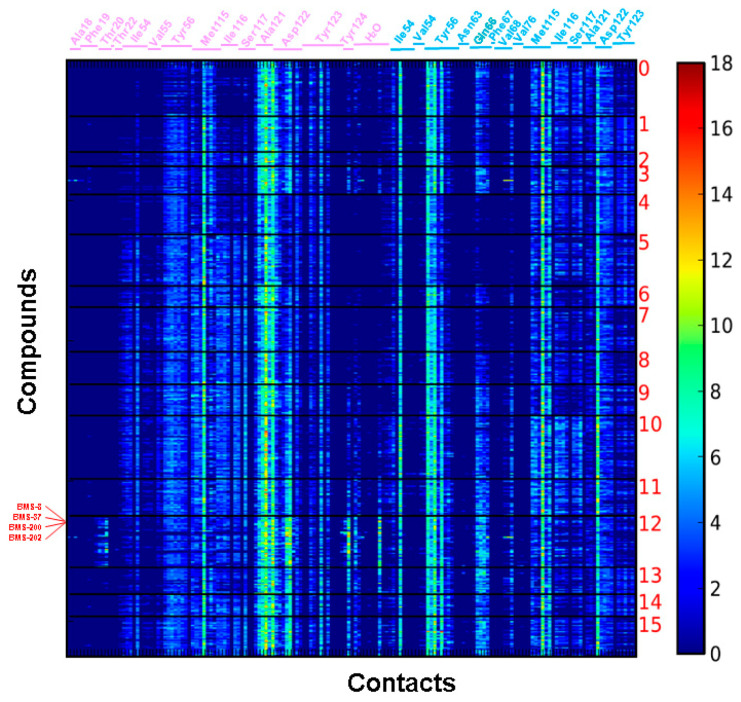
Contact fingerprints of 3929 candidates of the PD-1/PD-L1 inhibitor with idock scores less than −9.95 kcal/mol were analyzed by AuPosSOM. These candidates were classified into 16 groups according to their binding modes and strengths. Each row defines the contact fingerprint of each chemical structure to the interaction atoms of PD-L1. The color scale on the right shows the strength of interaction. Four known PD-1/PD-L1 inhibitors were also included in the analysis as the contact reference, and their names are labeled in red. Amino acids involved in the contact of each subunit of the PD-L1 dimer are labeled in plum (chain C) and light blue (chain D), respectively. The numbers of amino acids in each chain are labeled following NCBI reference sequence NM_014143.4.

With a large grid box used in the virtual screening, contact fingerprint analysis was used to classify the 3929 candidate structures according to their interaction mode with the PD-L1 dimer using the AuPosSOM (automatic analysis of poses using self-organizing maps) web server [15]. Known PD-1/PD-L1 inhibitors, BMS-8, BMS-37, BMS-200, and BMS-202, were also included in the analysis as the positive references for clustering. The results are shown in Figure 2. Although a large grid box was used in the virtual screening, the docking sites of these candidates in their best docking poses are quite localized, probably due to the limited space available within the PD-L1 dimer. Most of these candidates contact with amino acid residues like BMS-8, BMS-37, BMS-200, and BMS-202 with minor differences in contact strength and interaction residues. BMS-8, BMS-37, BMS-200, and BMS-202 are clustered together into group 12, which contains 368 chemical structures and bears the most intense contact fingerprint among all groups. The 368 chemical structures have averaged the molecular weight of 419.71 Da; hydrogen bond donors, 1.62; hydrogen bond acceptors, 6.32; and xlogP, 4.18. Compounds within this group were selected for downstream analysis.

### 2.3. Drug-Likeness Properties Filtering and Structure Clustering

**Figure 3:**
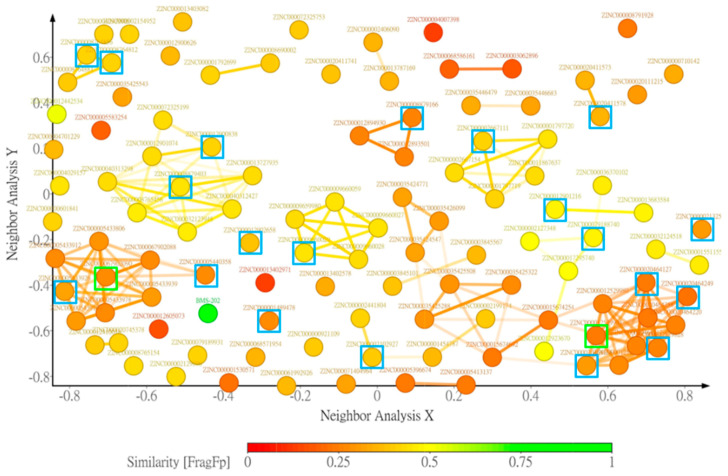
Similarity and clustering analysis of 111 candidates. The candidates were compared to the known PD-L1 dimer inducer, BMS-202 (green dot), and clustered according to substructure fragment (FragFP) similarity using DataWarrior. Colors of the candidates are marked according to the similarity scale relative to BMS-202. The dots enclosed in squares represent compounds selectively evaluated using the AlphaLISA PD-1/PD-L1 binding inhibition assay, and green boxes and blue boxes label the compounds capable and incapable of inhibiting the PD-1/PD-L1 interaction, respectively.

To eliminate compounds with unfavorable pharmacological properties that could lead to experimental animals suffering in downstream in vivo testing or early termination of the clinical trial, the 368 candidates in contact fingerprint group 12 were further evaluated and filtered according to the criteria mentioned in the Materials and Methods. A total of 111 compounds passed these criteria and were further clustered according to the FragFP descriptor with a minimum similarity of 0.8; and 56 clusters were generated with different numbers of compounds in each group (Figure 3 and Supplementary Materials Table S1). There were nine clusters with more than three compounds and six clusters with more than five compounds.

### 2.4. PD-1 and PD-L1 Binding Inhibition Assay

**Figure 4:**
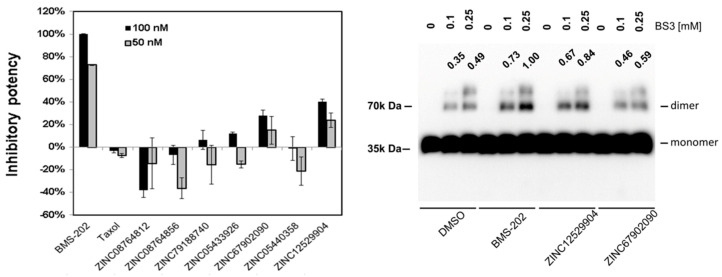
Effects of selected candidates on the PD-1/PD-L1 interaction and PD-L1 dimer formation. Activity of selected candidates were tested using the PD-1/PD-L1 binding inhibition assay (**left** panel) and the PD-L1 dimer formation assay with the protein crosslinking reagent (**right** panel). The extent of PD-1/PD-L1 binding and amount of dimer formation induced by 100 nM of BMS-202 were defined as 100%. The relative intensities of the PD-L1 dimer bands in the Western blot result were quantitated using ImageJ [16] and are shown in the right panel.

Twenty-two compounds with higher docking scores were purchased to evaluate their effects on the PD-1/PD-L1 interaction using the AlphaLISA PD-1/PD-L1 binding assay at concentrations of 50 nM and 100 nM. These compounds were incubated with the PD-L1 recombinant protein to induce dimer formation, followed by addition of the PD-1 protein to compete for the PD-1/PD-L1 interaction. The magnitude of BMS-202 on the reduction of the PD-1/PD-L1 interaction was defined as 100%. The results are shown in the left panel of Figure 4, and two of them, ZINC67902090 and ZINC12529904, have the potencies of 30 to 40% for inhibiting the PD-1/PD-L1 interaction, while some others, such as ZINC08764812 and ZINC08764856, could enhance the PD-1/PD-L1 interaction.

### 2.5. PD-L1 Dimer Formation Assay

**Figure 5:**
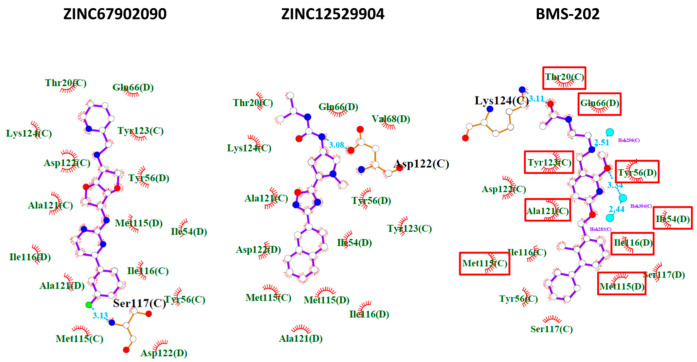
2D interaction maps between inhibitors and the PD-L1 dimer. The predicted potential interactions between inhibitors and the PD-L1 dimer were analyzed using LigPlot+ [17]. Hydrogen bonds between inhibitors and PD-L1 are shown in blue dotted lines labeled with distance in angstroms. Amino acid residues of PD-L1 involved in the hydrophobic interactions between inhibitors are labeled with eyelashes pointing to the interacted functional moiety of inhibitors. White, red, blue, green, and light blue circles represent carbon, oxygen, nitrogen, fluoride, and water molecules, respectively. Common amino acid residues of PD-L1 involved in the interactions with the three compounds were highlighted with red rectangles.

To test whether ZINC67902090 and ZINC12529904 inhibited PD-1/PD-L1 binding through inducing PD-L1 dimer formation, a crosslinking experiment was conducted. The protein complex formation in the absence or presence of inhibitors was crosslinked by the BS3 crosslinker and analyzed using the immunoblot analysis. As shown in the right panel of Figure 4, the addition of the positive control, BMS-202, significantly induced PD-L1 dimer formation compared with the control group. Both of the two identified PD-1/PD-L1 inhibitors promoted PD-L1 dimer formation like BMS-202, and the amount of the PD-L1 dimer was more significantly induced by ZINC12529904- and was only slightly increased by ZINC67902090. The extent of PD-L1 dimer formation induced by the three compounds followed the ranking of their potencies in the inhibition of the PD-1/PD-L1 interaction. The predicted potential interactions between ZINC67902090, ZINC12529904, and BMS-202 and the PD-L1 dimer involved at least one hydrogen bond and many hydrophobic interactions with a minimum of 13 amino acid residues. BMS-202 was bound to PD-L1 with additional hydrogen bonds with cocrystal water molecules. Nine amino acid residues of PD-L1 were shared in interactions with the three compounds. The detailed predicted potential interaction modes are illustrated in Figure 5.

## 3. Discussion

Immunotherapy using monoclonal antibodies has emerged as an ultimate hope for many advanced-stage cancer patients and has successfully extended their life and even made cancer completely disappear for many formerly incurable patients. Despite of this advance, a low overall response rate due to immunogenicity and low tissue penetrance of monoclonal antibodies [18] has further driven the quest for better therapeutic agents to benefit more patients. Currently, several small molecular inhibitors disrupting the PD-1/PD-L1 interaction that could potentially overcome these limitations of therapeutic antibodies have been identified, including several biphenyl-based derivatives [19,20,21], aromatic acetylene, and aromatic vinyl derivatives [22], as well as several other inhibitors without structure disclosure, such as CA-170 [23]. All these inhibitors are still in the early stage of development. Further structure optimization and continuous clinical development of these compounds are still needed to make small molecular PD-1/PD-L1 inhibitors available in clinical practice.

Ethnomedicine provides rich information for new drug discovery. Many natural products have been employed to regulate immune activities [24], but whether their immune-modulating properties could be through regulating the PD-1/PD-L1 interaction has not been investigated. In the current study, high throughput virtual screening was harnessed to identify potential small molecular PD-1/PD-L1 inhibitors using a 180,000 natural compound dataset from the ZINC12 database. Of the 111 compounds having docking scores comparable or better than BMS-202, 22 with similar contact fingerprints and preferable drug properties were chosen for in vitro evaluation. Two of them, ZINC67902090 and ZINC12529904, were able to inhibit the PD-1/PD-L1 interaction in the PD-1/PD-L1 AlphaLISA binding assay, albeit only exhibiting 30~40% inhibitory potencies compared with BMS-202. In the subsequent crosslinking assay, ZINC12529904 was found capable of promoting PD-L1 dimer formation significantly like BMS-202, while ZINC67902090 showed the ability only slightly. The difference in the extent of inhibiting the PD-1/PD-L1 interaction and promoting PD-L1 formation by ZINC6702090 might be due to a different buffer system used in the PD-1/PD-L1 binding assay (50 mM Tris (pH = 7.4), 0.015% TritonX-100, and 0.1% bovine serum albumin (BSA)) and the crosslinking assay (137 mM NaCl, 2.7 mM KCl, 4.3 mM Na_2_HPO_4_, 1.47 mM KH_2_PO_4_), as different compositions and concentrations of solutes in a buffer could affect the exposure of the hydrophilic and hydrophobic surfaces of proteins and the downstream protein-protein interaction. ZINC12529904 belongs to the biggest cluster group among the 111 selected candidates for in vitro screening, which are mostly in linear shape with a pyrrolidine-oxadiazole core in the middle, and benzene, naphthalene, or diphenyl derivatives on either side. Although it is too early to make any conclusive prediction at this moment, according to the results of the five test compounds in this cluster group, a bigger hydrophobic moiety composed of bicyclic aromatic ring structures in the oxadiazole side and a smaller moiety with more hydrophilic decoration in the pyrrolidine side may be required to stabilize the PD-L1 dimer structure and improve the potency. Although a strong small molecular PD-1/PD-L1 inhibitor was not identified, the current results suggested that more structurally diverse small molecules may be able to regulate the PD-1/PD-L1 interaction and should warrant expansion of the screening scope for discovering more PD-1/PD-L1 inhibitors of a small molecular weight.

Shielding the PD-1/PD-L1 binding interface of PD-L1 could play an important role in the regulation of the T cell response; and recent findings have shown that CD80 could form a heterodimer with PD-L1 by binding to the same interaction interface of the PD-L1 dimer to block the PD1-/PD-L1 interaction [25]. Although it is not known how many PD-L1 dimers are on the cellular surface and whether the PD-L1 dimer affects the in vivo activity of T cells in the physiological condition, uncoupling these types of PD-L1 complexes may help optimize the T cell response and even overt T cell hyperactivation. In the search for compounds to inhibit the PD-1/PD-L1 interaction by stabilizing the PD-L1 dimer, compounds capable of promoting the PD-1/PD-L1 interaction were also identified (Figure 4). The docking pose may not always be in the lowest energy conformation, and the conformation energy difference over 25 kcal/mol between the bioactive conformer and the naturally occurring conformer was reported before [26]. Hence, validating whether a compound entering the PD-L1 dimer interspace transiently uncoupled the complex by conformation switch and promoted the PD-1/PD-L1 interaction may require a long molecular dynamic simulation. It could be anticipated, however, that compounds capable of disrupting the PD-L1/CD80 complex to help optimize the T cell function could also be identified following the same scenario. If the PD-1/PD-L1 interaction enhancers also -exist in natural products, understanding the content and distribution of these PD-1/PD-L1 interaction suppressors and enhancers within natural products would be critical for public health. Unfortunately, these messages are not found in either the ZINC database, the commercial distributors of these compounds, or any other natural product databases [27], such as NaprAlert [28] and Super Natural II [29]. More comprehensive chemoinformatic collection of related information would still be required to make this information more easily accessible to researchers and the public.

In summary, natural products provide a plethora of information for new drug discovery, and two new PD-1/PD-L1 lead inhibitors with novel structure were efficiently and cost-effectively identified from over 180,000 natural products using virtual screening. The present results also indicated that both PD-1/PD-L1 inhibitors and enhancers are potentially present in natural products. Expansion of the screening scope and further structure optimization could eventually identify useful immune modulators to help improve public health.

## 4. Materials and Methods

### 4.1. Protein Structure Processing and Ligand Dataset Preparation

PDB file of the PD-L1 protein structure was processed by repairing errors using PDBfixer [30], assigning the protonation state at pH = 7.0 using PROPKA [31] and processing to the pdbqt format for docking using AutoDockTools [32]. A natural compound dataset, ZBC, containing 180,131 chemical structures in the mol2 format was downloaded from the ZINC12 database and converted into the pdbqt format using AutoDockTools without performing any cleanup steps in conversion.

### 4.2. Virtual Screening

Molecular docking and screening were conducted using the CUDA-accelerated version of the AutoDock Vina program [33], idock [34] under the Ubuntu 16.04 LTS system. A grid box encompassing amino acid side chains involved in the interactions between BMS-202 and chains C and D of the PD-L1 dimer protein crystal structure (PDB ID: 5J89) was used in the screening. The BMS-202-depleted protein with cocrystal water molecules was converted into the pdbqt format by AutoDockTools for docking screening. The grid box was of dimensions 24.5 Å × 21.5 Å × 28.25 Å with the center at 11.583 Å, 29.972 Å, and 183.917 Å. Nine docking poses were generated for each chemical structure in the flexible docking approach, and the score for the best docking pose of each chemical structure was used for ranking. The top-ranking candidates were further classified according to their contact modes and strengths with the PD-L1 dimer using the AuPosSOM 2.1 web interface (https://www.biomedicale.univ-paris5.fr/aupossom/) [15]. The compounds clustered together with known inhibitors, including BMS-8, BMS-37, BMS-200, and BMS-20, were chosen for further refinement.

### 4.3. Drug Likeness Prediction and Clustering Analysis

Criteria for predicting drug likeness properties with DataWarrior included molecular weight, 55–500 Dalton; hydrogen bond donors, ≤5; hydrogen bond acceptors, ≤10; octanol-water partition coefficient logP, −1–5; net charge, −2–2 (according to the -Lipinski’s rule of five) [35,36]; topological polar surface area, <100; no risks of mutagenicity and tumorigenicity; and no irritating and reproductive effects [37].

### 4.4. PD-1/PD-L1 Binding Inhibition Assay

The PD-1/PD-L1 binding inhibition assay was conducted using the PD-1/PD-L1 AlphaLISA binding assay (PerkinElmer, Houston, TX, USA) according to the manufacturer’s protocol. Initially, the PD-L1 protein was incubated with small molecular inhibitor candidates for 2 h, followed by addition of the PD-1 protein into the mixture to compete with putative inhibitors for PD-L1 binding for 1.5 h. After incubation, acceptor beads and donor beads were added into the reaction mixture to interact with PD-1 and PD-L1, respectively. The influence of these inhibitor candidates on the PD-1/PD-L1 binding was measured using PerkinElmer Enspire X1 with the absorbance value of 615 nm after the 680 nm excitation. All experimental procedures were conducted at 25 °C using a thermal incubator.

### 4.5. Crosslinking Assay

Effects of these candidates on PD-L1 dimer formation were analyzed using the protein crosslinking assay. In brief, 10 μM of His-tag hPD-L1 were incubated with the equal molar concentration of candidate inhibitors in the PBS buffer at 25 °C for 1 h, followed by incubation with a BS3 crosslinking reagent (Cayman, Ann Arbor, MI, USA) at the final concentrations of 0.1, 0.25 mM for 30 min at 25 °C in a thermal incubator. At the end of incubation, the crosslinking reaction was quenched by the addition of the Tris buffer (pH 7.5) to the final concentration of 25 mM. The effects of the candidate inhibitors on PD-L1 dimer formation were evaluated by resolving the crosslinking products with discontinuous SDS-PAGE and transferring onto a polyvinylidene fluoride (PVDF) membrane. The PD-L1 protein signals on the membrane were detected using an anti-His tag antibody (1:1000, cat. sc-8036, Santa Cruz, Dallas, TX, USA) as visualized with horseradish peroxidase (HRP)-conjugated goat anti-mouse secondary antibody (1:5000, cat. 12349, Merck, Burlington, MA, USA) and the Immobilon Western Chemiluminescent HRP Substrate (cat. WBKLS0500 Merck) and recorded using the Syngene G:BOX chemi XX9 gel imaging system (Syngene, Cambridge, UK).

## Supplementary Materials

The following are available online: Table S1: The docking scores, chemical properties, and clustering information of the selected 111 compounds.
